# Identifying the genetic association between severe autoimmune type 2 diabetes and the risk of focal epilepsy

**DOI:** 10.3389/fendo.2024.1396912

**Published:** 2024-11-06

**Authors:** Huanhua Wu, Kai Liao, Ying Li, Zhiqiang Tan, Ziqing Zhou, Chunyuan Zeng, Jian Gong, Huadong Wang, Hao Xu, Youzhu Hu

**Affiliations:** ^1^ Central Laboratory, The Affiliated Shunde Hospital of Jinan University, Foshan, Guangdong, China; ^2^ Department of Nuclear Medicine, The First Affiliated Hospital of Chongqing Medical University, Chongqing, China; ^3^ Department of Nuclear Medicine and PET/CT-MRI Center, The First Affiliated Hospital of Jinan University and Institute of Molecular and Functional Imaging, Jinan University, Guangzhou, Guangdong, China; ^4^ Department of Pharmacology, Medical College of Jinan University, Guangzhou, Guangdong, China; ^5^ Department of Nuclear Medicine, Nanhai District People's Hospital of Foshan, Foshan, Guangdong, China; ^6^ Department of Pathophysiology, Key Laboratory of State Administration of Traditional Chinese Medicine of the People's Republic of China, School of Medicine, Jinan University, Guangzhou, Guangdong, China; ^7^ Department of Hepatobiliary Surgery, The First Affiliated Hospital, Jinan University, Guangzhou, Guangdong, China

**Keywords:** severe autoimmune type 2 diabetes, focal epilepsy, two sample Mendelian randomization, causal associations, GWAS

## Abstract

**Background:**

Observational studies suggested a bidirectional relationship between severe autoimmune type 2 diabetes and focal epilepsy. However, it remains debated whether and in which direction a causal association exists. This genetics-based study aimed to explore the relationships of severe autoimmune type 2 diabetes (T2DM) and focal epilepsy outcomes with two sample Mendelian randomization (TSMR) method.

**Methods:**

Genetic instruments were obtained from large-scale genome-wide meta-analysis of severe autoimmune T2DM (Ncase = 452, Ncontrol = 2,744), and focal epilepsy (Ncase = 929, Ncontrol = 212,532) of European ancestry. A series of analyses were performed to select eligible genetic instruments robustly associated with each of the traits using summary-level statistics. Inverse variance weighted was used for primary analysis, with alternative 11 MR methods. A scatter plot was utilized to illustrate the association between single nucleotide polymorphism (SNP) effects on the exposure and SNP effects on the outcome. The Wald ratio for individual SNPs and their cumulative effects was depicted using a forest plot. And diagnostics and sensitivity analyses were used to evaluate if the causal estimates are robust to violations of MR underlying assumptions, including pleiotropy, heterogeneity assessment, and leave-one-out analysis. Then the results were validated using CURATED database of DisGeNET platform.

**Results:**

For forward analysis, genetic predisposition to severe autoimmune T2DM was associated with an increased risk of focal epilepsy (Inverse variance weighted (IVW) method: OR = 1.11, 95% CI = 1.03-1.18, *p* = 0.012). For reverse analysis, there was no enough instrument variables of focal epilepsy on severe autoimmune T2DM. Further, the interrelation between severe autoimmune T2DM and focal epilepsy was demonstrated via variant-disease association network analysis using the instrument SNPs.

**Discussion:**

This MR study supports a causal link between severe autoimmune T2DM and focal epilepsy. More effort should be made to screen seizure in severe autoimmune T2DM, unravel its clinical implications, and explore its role as a putative modifiable risk factor.

## Introduction

Emerging evidence suggests a compelling interconnection between severe autoimmune type 2 diabetes mellitus (T2DM) and focal epilepsy, paving the way for a deeper understanding of their complex relationship. Insights from preclinical studies have unveiled a deleterious reciprocal mechanism between type 2 T2DM and epileptic seizures (1). The coexistence of epilepsy and T2DM is frequently observed, and obesity has been recognized as a shared risk factor for both conditions. Remarkably, individuals with epilepsy exhibit reduced physical activity levels, thereby compromising the protective effects against obesity. Pertinent to both epilepsy and T2DM, impaired mitochondrial function and inadequate adiponectin levels play a prominent role (2). Moreover, a myriad of additional factors may contribute to the heightened susceptibility to seizures in individuals with T2DM, encompassing imbalances in excitatory and inhibitory neurotransmission, neuroinflammation, and metabolic alterations.

In the realm of human clinical studies, association between diabetes mellitus and epilepsy has been unveiled through comprehensive epidemiological investigations (1, 3, 4). Multiple studies have consistently reported this reciprocal relationship, elucidating its prevalence. A recent population-based study revealed a noteworthy 1.44-fold augmented risk of developing epilepsy in individuals affected by T2DM (1). However, it is crucial to acknowledge the inherent limitations of these retrospective investigations, as they are susceptible to confounding effects stemming from cerebrovascular insults, education levels, and the varied use of antiepileptic medications, which can exert diverse effects on neurological disorders. Moreover, the validity of these studies is restricted due to small sample sizes and the potential for confounding variables and reverse causality.

Given the close interconnection between severe autoimmune T2DM and focal epilepsy, it is imperative to establish a definitive causal association, as such an association would highlight a modifiable cause or a previously overlooked consequence of focal epilepsy. However, there is an ongoing debate regarding whether epilepsy leads to focal epilepsy or vice versa (5). In contrast to a causal relationship, it is also plausible that both conditions share a common underlying mechanism (such as neuroinflammation and neurovascular unit dysfunction). Moreover, retrospective studies may be constrained by the potential confounding effects. For instance, certain antiepileptic drugs, which might have a negative impact on cognition, are likely to contribute to the observed correlation between severe autoimmune T2DM and focal epilepsy.

Mendelian randomization (MR) applies genetic variants that exhibit robust associations with exposure as instrumental variables to ascertain the causal impact of a suspected exposure on the outcome. When the requisite assumptions are met, the MR technique mitigates numerous inherent limitations inherent in conventional observational studies, such as unobserved confounding and reverse causality (6).

In this investigation, we embraced a two sample MR approach to examine the relationship between severe autoimmune T2DM and focal epilepsy. We further explored the potential associations between candidate single-nucleotide polymorphisms (SNPs) and relevant diseases.

## Methods

### Data sources

This study is based on publicly accessible, summary-level genome-wide association study (GWAS) data. The summary statistics for severe autoimmune T2DM, and focal epilepsy did not contain any personal information, and the GWAS have obtained ethical approval from relevant ethics review boards. Through consistent research findings, T2DM has shown an observable clustering pattern, resulting in several distinct subtypes characterized by unique disease progression trajectories, varying risks of complications and partially distinct genetic backgrounds. Severe autoimmune T2DM summary statistics are available in the GWAS Catalog (www.ebi.ac.uk/gwas/) under accession no.GCST90026412 (7) (Ncase = 452, Ncontrol = 2,744, number of SNPs = 5,376,535). Of the participants, all had European Scandinavian ancestry. Severe autoimmune diabetes (6% of patients) were defined by the presence of glutamic acid decarboxylase autoantibodies with relatively low BMI, poor metabolic control, and insulin deficiency (8).

For the study on focal epilepsy, the summary statistics for the GWAS were acquired from the Medical Research Council Integrative Epidemiology Unit (IEU) Open GWAS project (https://gwas.mrcieu.ac.uk/datasets/finn-b-FE/) (Ncase = 929, Ncontrol = 212,532, number of SNPs = 16,380,452). Of the participants from FinnGen biobank analysis round 5, all had European ancestry. The FinnGen study is a large-scale genomics initiative that has analyzed over 500,000 Finnish biobank samples and correlated genetic variation with health data to understand disease mechanisms and predispositions. The project is a collaboration between research organizations and biobanks within Finland and international industry partners (9). Focal epilepsy was determined with ICD-10: G403 and ICD-8: 3453. Detailed information about the focal epilepsy subjects, endpoint definition, and statistical analysis have been described in the homepage of FINNGEN: https://r5.risteys.finngen.fi/phenocode/FE.

The MR investigation exclusively utilized published or publicly accessible GWAS data. Informed consent was procured from all participants involved in the initial genome-wide association investigations, as detailed in the original publications and consortiums.

### Instrument selection

For the genetic instruments used in our TSMR analysis of severe autoimmune T2DM and focal epilepsy, we selected single-nucleotide polymorphisms (SNPs) that demonstrated a strong association with each respective exposure, applying a stringent significance threshold (*p* < 5 × 10^-8^) and an *F*-statistic greater than 10 to ensure robust instruments. To manage linkage disequilibrium (LD), we employed LD-based clumping with an *r*² threshold of 0.01 and a maximum distance threshold of 5,000 kilobases. This procedure ensured the selection of independent SNPs, thereby minimizing the risk of confounding due to correlated variants. Standard quality control procedures were implemented, including the assessment of imputation info score, call rate, Hardy-Weinberg equilibrium, and heterogeneity. We also applied Steiger filtering to confirm that the selected variants exhibited a stronger association with the outcome (focal epilepsy) than with the exposure (severe autoimmune T2DM) (10).

SNPs that were absent in the outcome dataset and lacked appropriate proxy SNPs were excluded from the analysis. We harmonized the remaining SNPs by aligning allele frequencies and ensuring that effect alleles were consistent between the exposure and outcome data. Non-biallelic and palindromic SNPs (A/T, G/C) were excluded unless strand ambiguity could be resolved. Additionally, SNPs with a minor allele frequency (MAF) of less than 0.01 were excluded to avoid potential low-confidence results. The final set of SNPs included in our analysis was carefully selected based on the criteria mentioned above. We prioritized SNPs that were both statistically significant and independent of other variants, ensuring a robust and unbiased set of instruments for the TSMR analysis. The use of stringent selection criteria and thorough quality control measures contributes to the validity and reproducibility of our findings.

### MR assumptions and sensitivity analyses

**Figure 1:**
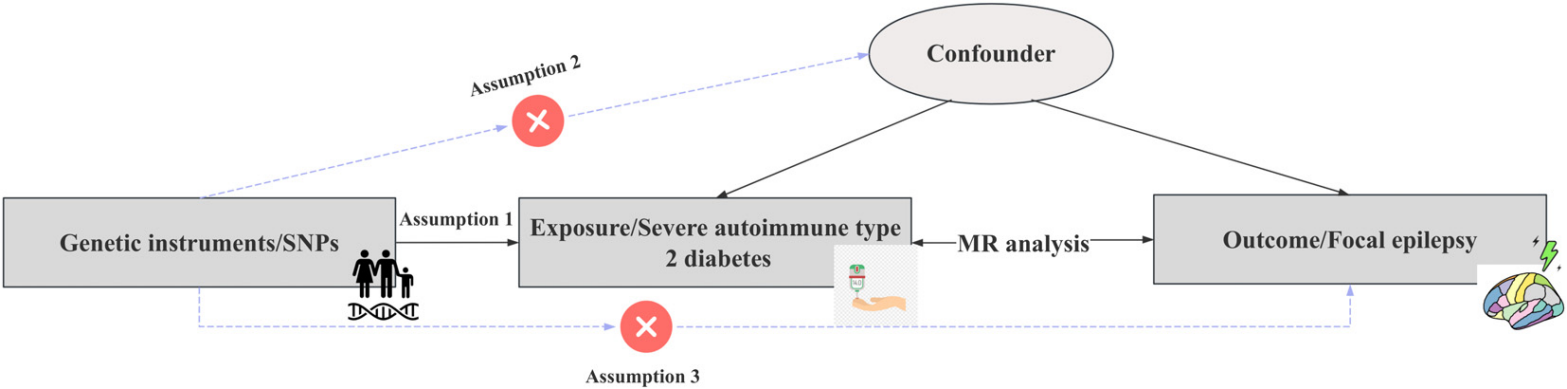
Schematic representation of the study’s design and procedural steps. To substantiate the presence of a causal relationship, specific conditions need to be satisfied: Assumption 1): the instrumental variables exhibit an association with the Severe autoimmune T2DM. Assumption 2): the instrumental variables are not associated with confounding factors. Assumption 3): the instrumental variables do not possess a direct connection with the Focal epilepsy.

We conducted a two-sample Mendelian randomization (TSMR) investigation to examine the causal relationships between severe autoimmune T2DM and focal epilepsy using data from the GWAS repository. The MR approach in our study adhered to three key assumptions, as illustrated in **Figure 1**. All instrumental variables used in our analyses demonstrated an *F*-statistic greater than 10, indicating minimal risk of weak instrument bias. Sensitivity analyses were performed, incorporating SNPs that passed the heterogeneity in Radial regression analysis (11). Twelve various techniques, including Maximum likelihood, MR Egger, MR Egger (bootstrap), Simple median, Weighted median, Penalized weighted median, Inverse variance weighted (fixed effects), Inverse variance weighted, Simple mode, Weighted mode, Weighted mode (NOME), and Simple mode (NOME) methods, were employed. Each method relied on distinct underlying assumptions.

To assess the impact of individual variants on the MR results, we conducted leave-one-out analyses, visualized through leave-one-out plots. Cochran’s Q statistic was used to detect heterogeneity. We also generated forest and funnel plots to evaluate heterogeneity and pleiotropy using MR Egger and Inverse Variance Weighted methods, with MR-Pleiotropy RESidual Sum and Outlier (MR-PRESSO) method applied to identify and correct for pleiotropy and outliers. MR Egger regression was employed to detect horizontal pleiotropy via the Egger intercept. Robustness against outliers and invalid instruments was ensured using median-based and mode-based methods. For cases with only one SNP as an instrument, the Wald ratio was reported.

### Variant-disease associations

To explore the molecular underpinnings of human diseases, we retrieved the information in DisGeNET for a list of variants identified by the NCBI Short Genetic Variations database identifiers. DisGeNET is an open access platform meticulously amalgamating diverse resources focused on genes and variants associated with human diseases, along with valuable insights extracted through meticulous text mining of the scientific literature (12). By searching multiple variants in the default source CURATED database (containing human gene-disease associations provided by the human expert curated data resources), we plotted the variant-disease association network, variant-disease class heatmap, and variant-gene-disease association network (13). Disease-associated SNPs generally do not implicate target genes, as most disease SNPs are regulatory (14). To identify autonomous significant SNPs and assign them to respective genes, we employed the disgenet2r R package, distinguishing genomic loci devoid of linkage disequilibrium.

### Statistical analysis

The main analyses were performed with R software (version 4.3.1) using the TwoSampleMR (15) (version 0.5.7), Mendelian Randomization (16) (version 0.9.0), MRPRESSO (17) (version 1.0), RadialMR (11) (version 1.1), and disgenet2r (12) (version 0.99.3) packages. We adhered to the Strengthening the Reporting of Observational Studies in Epidemiology using MR (Mendelian randomization) guideline to present our results (18). All hypothesis testing was carried out using a 2-sided approach, with a significance threshold set at *p* < 0.05. Codes are available on reasonable request.

## Results

### Effects of T2DM on focal epilepsy

**Figure 2:**
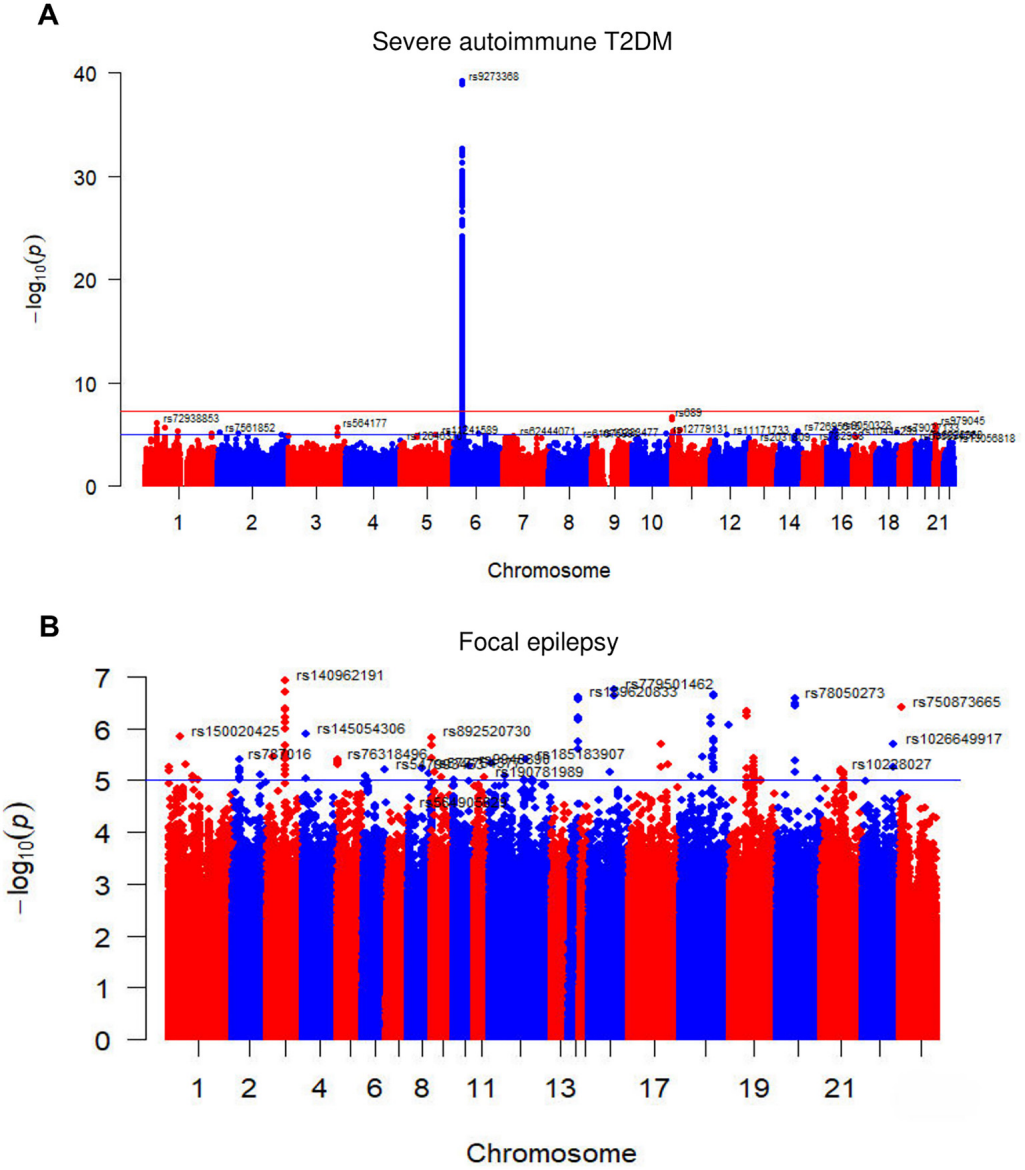
The Manhattan plots of GWAS results of the **(A)** Severe autoimmune T2DM and **(B)** Focal epilepsy. The x-axis represents the SNPs locus on the chromosome, and the y-axis represents the *p* value of the SNPs locus. The red and blue reference lines represent -log_10_ logarithm of *p* values of 5 10^-8^ and 1 10^-5^, respectively. T2DM = type 2 diabetes.

**Figure 3:**
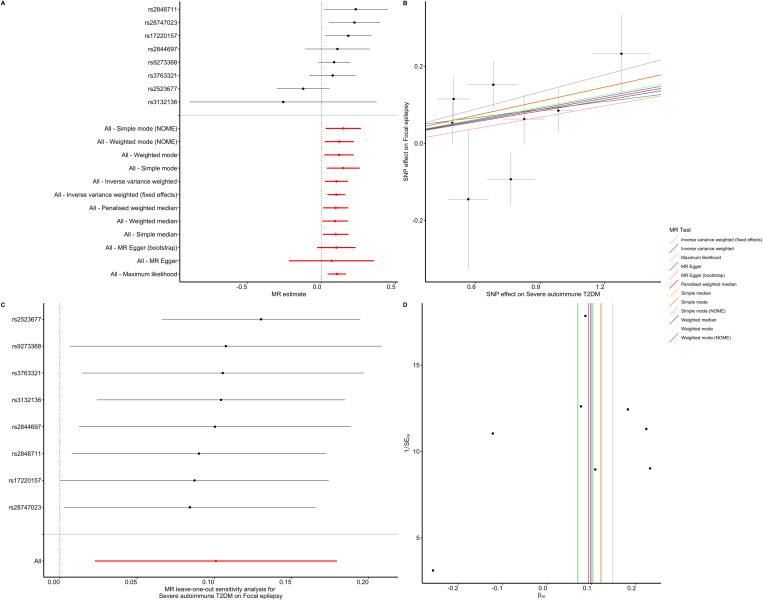
Association between severe autoimmune T2DM and focal epilepsy estimated by the TSMR. **(A)** Forest plot of focal epilepsy for each 1 SD increase of severe autoimmune T2DM risk. **(B)** Scatter plot showing the effect of genetic instruments on severe autoimmune T2DM risk against their effect on focal epilepsy according to 12 methods. **(C)** There was no substantial change of IVW causal estimate after removing any of the instrumental SNPs. **(D)** The funnel plot showed no asymmetry according to 12 methods.

**Table 1** presents a comprehensive overview of the key characteristics pertaining to the contributing genome-wide association studies (GWAS). The Manhattan plots depicted in **Figure 2** were used to display the relationship between all loci and their corresponding *p* values for the 22 chromosomes; the blue reference line represents the *p* value threshold of 1 × 10^-5^. Eight single nucleotide polymorphisms (SNPs) associated with severe autoimmune T2DM in the context of focal epilepsy reached genome-wide significant levels. These SNPs (rs17220157, rs2523677, rs2844697, rs2848711, rs28747023, rs3132136, rs3763321, and rs9273368) were strongly linked to severe autoimmune T2DM (*p* < 1 × 10^-8^) according to **Table 2**. The impact of severe autoimmune T2DM on focal epilepsy is illustrated in **Figure 3**. Notably, individuals with a genetic predisposition to severe autoimmune T2DM exhibited an increased risk of developing focal epilepsy, with an odds ratio (OR) of 1.11 (95% CI = 1.03-1.18, *p*_IVW_ = 0.012) as shown in **Table 3**, **Figures 3A, B**. Extensive sensitivity analyses yielded consistent estimates in both magnitude and direction (*p*_Maximum likelihood_ = 0.001, *p*_Egger_ = 0.652, *p*_Egger (bootstrap)_ = 0.064, *p*_Simple median_ = 0.026, *p*_Weighted median_ = 0.030, *p*_Penalized weighted median_ = 0.021, *p*_IVW (fixed effects)_ = 0.001, *p*_IVW_ = 0.012, *p*_Simple mode_ = 0.035, *p*_Weighted mode_ = 0.054, *p*_Weighted mode (NOME)_ = 0.030, *p*_Simple mode (NOME)_ = 0.041). As no significant association between focal epilepsy and severe autoimmune T2DM was detected, the reverse MR analysis exploring the influence of focal epilepsy on severe autoimmune T2DM was not conducted.

**Table 1:** GWAS datasets enrolled in the Mendelian Randomization study.

Items	GWAS ID	Data source	Population	Sample size	Number of cases	Number of SNPs	Sex	Year
**Severe autoimmune T2DM**	ebi-a-GCST90026412	EBI database	European	3,196	452	5,376,535	Males and Females	2021
**Focal epilepsy**	finn-b-FE	FinnGen biobank	European	213,461	929	16,380,452	Males and Females	2021

**Table 2:** The information for final eight instrumental variables.

SNP	CHR	POS	EA	OA	EAF	β value	SE	*p* value
**rs17220157**	6	32787592	A	G	0.077	1.280	0.134	1.82 × 10^-21^
**rs2523677**	6	31434801	T	C	0.122	0.763	0.112	8.94 × 10^-12^
**rs2844697**	6	30932309	T	C	0.387	0.490	0.075	7.47 × 10^-11^
**rs2848711**	6	31385629	G	T	0.369	-0.496	0.076	7.07 × 10^-11^
**rs28747023**	6	32654807	A	G	0.136	-0.681	0.116	3.92 × 10^-9^
**rs3132136**	6	32853987	A	G	0.422	0.565	0.095	3.33 × 10^-9^
**rs3763321**	6	32406704	G	T	0.826	0.826	0.096	7.54 × 10^-18^
**rs9273368**	6	32626475	A	G	0.35	0.985	0.075	6.65 × 10^-40^

CHR, chromosome; POS, position; EA, effect allele; OA, other allele; EAF, effect allele frequency; SE, standard error.

**Table 3:** Causal effects of severe autoimmune T2DM on focal epilepsy outcome estimated by 12 methods.

Exposure	Outcome	Method	nSNP	OR (95% CI)	*p* value
**Severe autoimmune T2DM**	Focal epilepsy	Maximum likelihood	8	1.11 (1.05-1.17)	0.001
**Severe autoimmune T2DM**	Focal epilepsy	MR Egger	8	1.07 (0.79-1.36)	0.652
**Severe autoimmune T2DM**	Focal epilepsy	MR Egger (bootstrap)	8	1.11 (1.02-1.20)	0.064
**Severe autoimmune T2DM**	Focal epilepsy	Simple median	8	1.10 (1.02-1.19)	0.026
**Severe autoimmune T2DM**	Focal epilepsy	Weighted median	8	1.10 (1.01-1.18)	0.030
**Severe autoimmune T2DM**	Focal epilepsy	Penalised weighted median	8	1.10 (1.02-1.18)	0.021
**Severe autoimmune T2DM**	Focal epilepsy	IVW (fixed effects)	8	1.11 (1.05-1.17)	0.001
**Severe autoimmune T2DM**	Focal epilepsy	IVW	8	1.11 (1.03-1.18)	0.012
**Severe autoimmune T2DM**	Focal epilepsy	Simple mode	8	1.16 (1.05-1.27)	0.035
**Severe autoimmune T2DM**	Focal epilepsy	Weighted mode	8	1.13 (1.02-1.23)	0.054
**Severe autoimmune T2DM**	Focal epilepsy	Weighted mode (NOME)	8	1.13 (1.04-1.22)	0.030
**Severe autoimmune T2DM**	Focal epilepsy	Simple mode (NOME)	8	1.16 (1.04-1.28)	0.041

IVW, inverse variance weighted; NOME, NO Measurement Error.

The majority of our pleiotropy robust methods are significant, suggesting that the presence of severe autoimmune T2DM is associated with increased risk of focal epilepsy. Despite the lack of significance with the Egger method (*p*_Egger_ = 0.652, *p*_Egger (bootstrap)_ = 0.064, *p*_Weighted mode_ = 0.054), this approach accounts for horizontal pleiotropy at the expense of reduced statistical power and precision (19). Furthermore, the leave-one-out analysis bolstered these findings by revealing that the specific instrumental single nucleotide polymorphisms (SNPs) did not exert any influence on the causal effect (**Figure 3C**). The symmetry depicted in the funnel plot (**Figure 3D**) further indicated a negligible risk of directional pleiotropy.

### Sensitivity analysis

**Figure 4:**
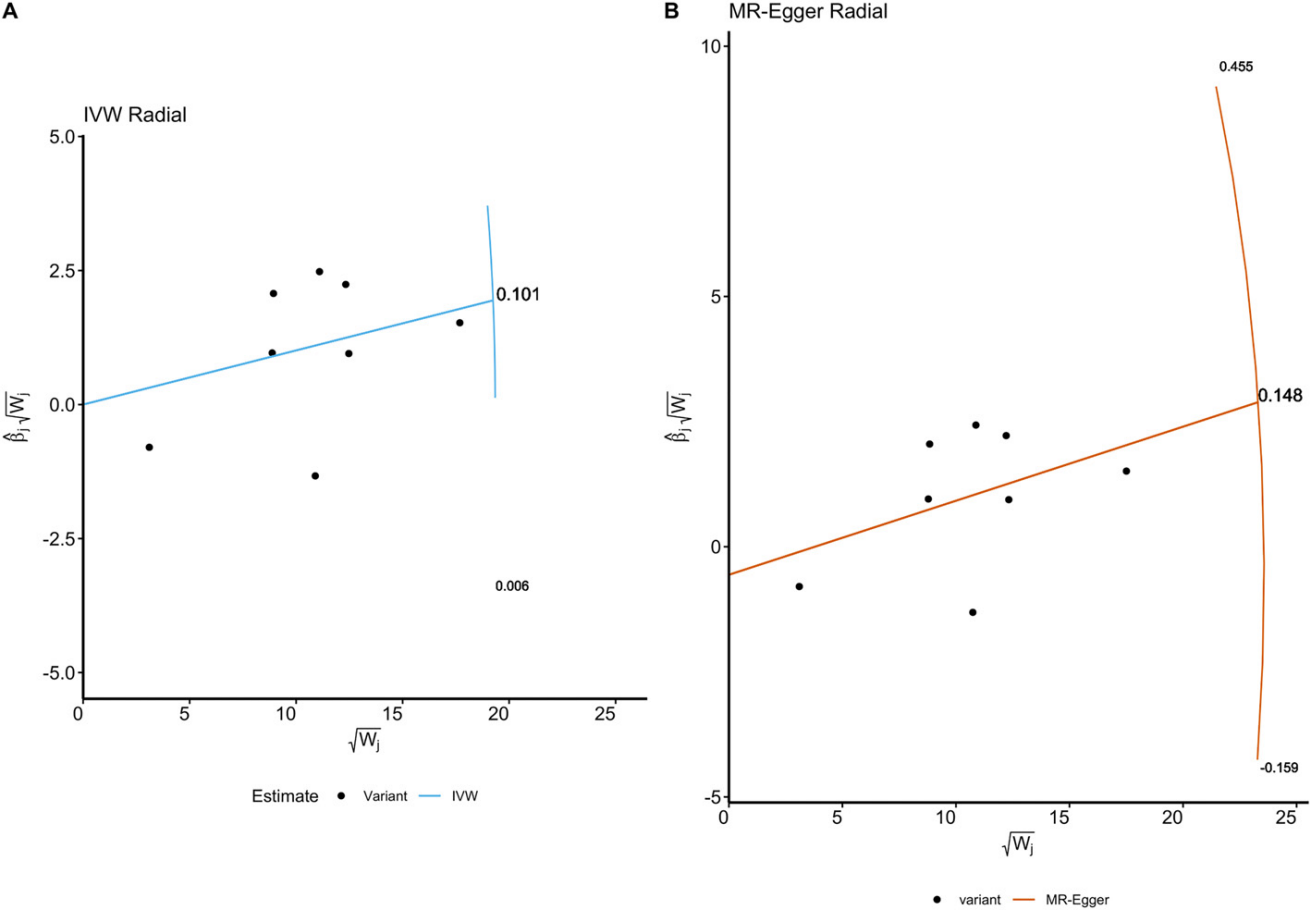
The Radial plots and Radial regression analysis for TSMR. **(A)** IVW Radial plot. **(B)** MR-Egger Radial plot. Both the IVW and MR Egger Radial MR methods identified no variants as potential outliers.

In the IVW analysis we observed that the existence of severe autoimmune T2DM was causally associated with an increased risk of focal epilepsy. And, the majority of our sensitivity analysis are significant suggesting that our IVW estimated is unbiased. Furthermore, the MR-Egger causal estimate is insignificant. The robustness of our causal estimate is guaranteed by the lack of significant heterogeneity, horizontal pleiotropy, and outliers (**Figures 3**, **4**).

As shown in **Table 4**, the MR-Egger intercept had a *p* value of 0.828, indicating that there was no significant deviation from zero, and thus no evidence of directional pleiotropy. The MR-PRESSO test yielded a *p* value of 0.157, also suggesting the absence of significant pleiotropic effects. Additionally, no outlier SNPs were identified by MR-PRESSO. Furthermore, the heterogeneity tests conducted using Cochran’s Q statistic showed no significant heterogeneity, with *p* values of 0.069 for MR-Egger and 0.107 for the Inverse Variance Weighted (IVW) method.

**Table 4:** Pleiotropy and heterogeneity assessment for significant results.

Directional pleiotropy		Heterogeneity		
Method	p *value*	Method	Q statistic	p value
**MR Egger intercept**	0.828	**MR Egger**	11.70	0.069
**MR-PRESSO**	0.157	**Inverse variance weighted**	11.80	0.107

These findings are further supported by the radial plots (**Figure 4**), which showed no variants classified as outliers under the IVW Radial MR and MR-Egger Radial MR methods. The lack of significant pleiotropy and heterogeneity in these tests suggests that our causal estimates are unlikely to be biased by pleiotropic effects. The absence of significant pleiotropy in our analyses supports the validity of the causal relationship observed between severe autoimmune T2DM and focal epilepsy.

### Variant-disease network

In UKBiobank ICD PheWeb platform (https://pheweb.org/UKB-SAIGE/) (20), rs17220157 (nearest gene: AL669918.1, HLA-DOB), rs2523677 (nearest gene: MICB), rs2844697 (nearest gene: SFTA2), rs2848711 (nearest gene: MICA), rs28747023 (nearest gene: HLA-DQB1), rs3132136 (nearest gene: PSMB9), rs3763321 (nearest gene: HLA-DRA), and rs9273368 (nearest gene: HLA-DQB1).

**Figure 5:**
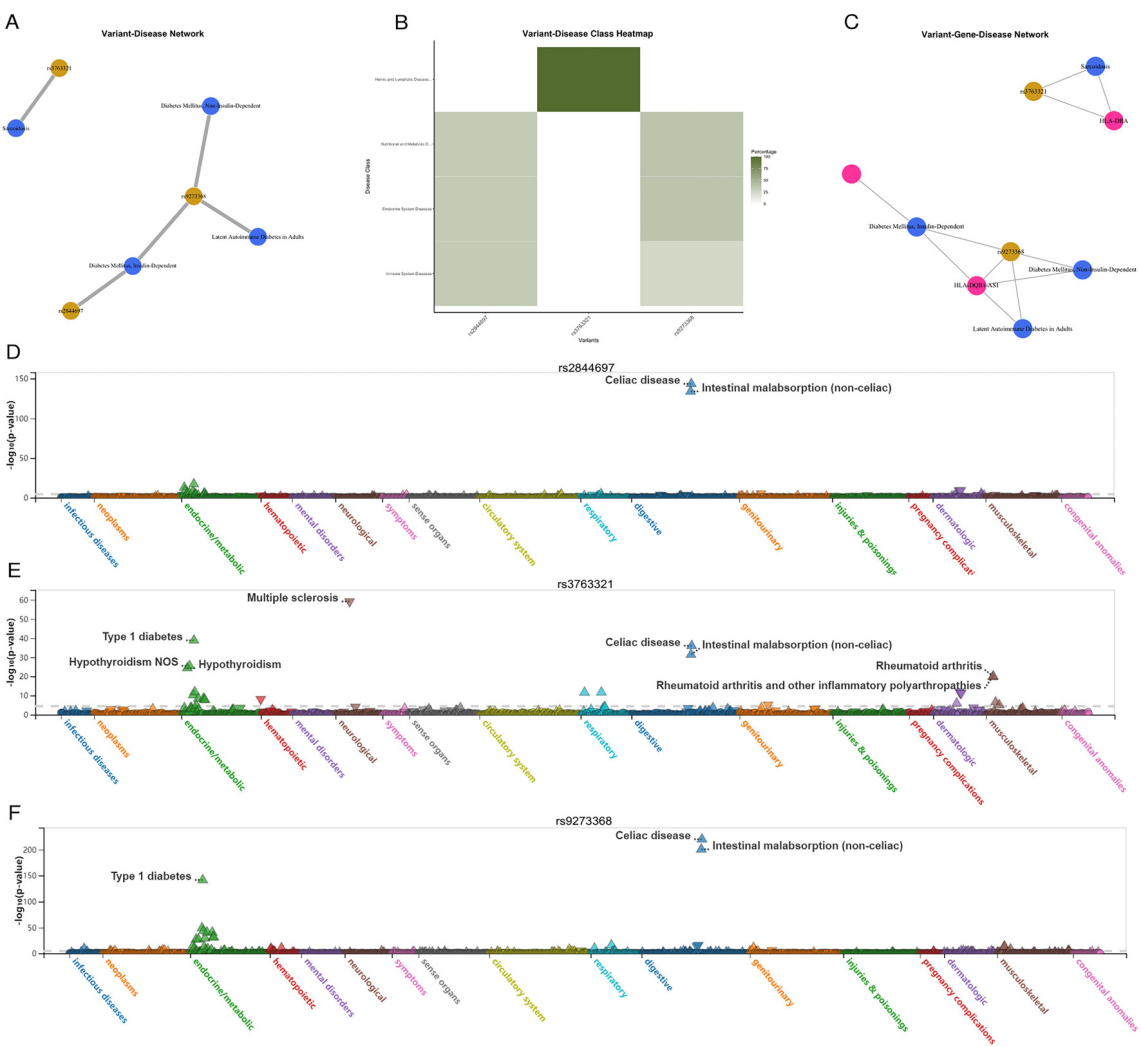
Human gene-disease associations and variant-disease associations from CURATED database of DisGeNET platform. **(A)** Variant-Disease Network. **(B)** Variant-Disease Class Heatmap. **(C)** Variant-Gene-Disease Network. **(D-F)** PheWAS view for variant rs2844697, rs3763321, and rs9273368. Traits are sorted andcolored according to a meaningful set of biological categories (e.g. celiac disease, intestinal malabsorption, metabolic). Direction of effect of the alternate allele’s association witheach trait is exhibited by upward-facing (positive effect) or downward-facing (negative effect) triangle.

According to the variant-disease network plots of mapped genes in GWAS Catalog were rs2844697, rs3763321, and rs9273368 (**Figures 5A-C**). Analysis of the final three overlapped selected variant using a phenome-wide view (PheWAS) revealed a significant association with a range of traits related to the urinary system or lower digestive tract, as identified in the UK Biobank. These traits have been systematically organized and color-coded based on relevant biological categories, such as infectious diseases, neurological traits, or metabolic traits. The effect of the alternative allele on each trait is depicted by upward-facing triangles for positive effects and downward-facing triangles for negative effects (21). **Figures 5D-F** presents various perspectives on genetic association signals and key variants related to severe autoimmune T2DM in the UK Biobank association results as depicted in the PheWeb. And the PheWAS view provides additional support for the association of this locus with a range of diseases, including celiac disease, intestinal malabsorption (non-celiac), multiple sclerosis, type 1 diabetes, hypothyroidism, and rheumatoid arthritis.

Interestingly, in the case of the other prominent loci (both on chromosome 6), the regional and PheWAS views (rs2844697 near SFTA2, rs3763321 near HLA-DRA, and rs9273368 near HLA-DQB1) clearly indicate that these loci exhibit associations with celiac disease and intestinal malabsorption (non-celiac). Although less well-studied, rs2844697 is associated with various traits related to the digestive and urinary systems. The variant’s involvement in conditions such as intestinal malabsorption and its proximity to immune-related loci suggest it may play a role in the broader network of autoimmune diseases. rs3763321 (HLA-DRA) is located near the HLA-DRA gene, which is involved in immune system functioning. Our analysis showed that rs3763321 is associated with several immune-related diseases, including sarcoidosis and type 1 diabetes. The PheWAS analysis further supports its involvement in autoimmune conditions, such as celiac disease and hypothyroidism, indicating a potential shared genetic pathway that may also influence the risk of focal epilepsy. Similarly, rs9273368 is located near the HLA-DQB1 gene, another key player in immune regulation. This SNP has been associated with both type 1 and type 2 diabetes, as well as latent autoimmune diabetes in adults (LADA). The overlap between these associations suggests that the genetic predisposition to autoimmune diabetes may extend to other autoimmune-related conditions, including focal epilepsy.

## Discussion

In this study, we have established a causal link between severe autoimmune T2DM and an increased risk of focal epilepsy, demonstrating the durability of this association through sensitivity analyses. Our study further reveals the causal connection between severe autoimmune T2DM and focal epilepsy, a relationship reinforced by sensitivity analyses and independently replicated using distinctive instrumental variables. While there exists evidence indicating a potential causal impact of severe autoimmune T2DM on focal epilepsy, our observations align with previous research (1, 22, 23). Significantly, research has revealed that individuals with a diagnosis of type 2 diabetes exhibit a 1.5-fold higher probability of developing epilepsy when compared to meticulously matched control individuals. Importantly, it has been observed that severe hypoglycemic episodes may contribute to an elevated risk of epilepsy; however, they do not modify the influence of type 2 diabetes in this context (1).

In our study, we utilized the DisGeNET platform to validate the findings from our Mendelian Randomization analysis by exploring the associations between the identified SNPs and a range of diseases (24). The variant-disease network analysis revealed significant associations for three key SNPs (rs2844697, rs3763321, and rs9273368) with various traits, particularly those related to the immune and endocrine systems. The variant-disease network analysis provides additional evidence that the SNPs identified in our study are biologically relevant and may contribute to the development of both severe autoimmune T2DM and focal epilepsy. The associations observed in the DisGeNET and PheWAS analyses support the hypothesis that shared genetic factors may underlie these two conditions, reinforcing the validity of our findings.

Previous study revealed markedly different genetic architectures between focal and generalized epilepsies (25). Prioritized candidate genes overlap with monogenic epilepsy genes and with targets of current antiseizure medications. A previous investigation underscored the advantages associated with the integration of diverse omics data in uncovering functional genes and potential regulatory mechanisms governed by local genetic variation. Prospective employment of collaborative omics data analyses is anticipated to enhance our comprehension of the biological pathways underlying T2DM and other prevalent medical conditions (25, 26). To date, GWASs have unveiled numerous loci linked to immune-related disorders (27). A recent investigation revealed concurrent links between disease susceptibility and blood immune cell-associated signals, highlighting intermediary quantitative traits that help bridge the gap between genetic variability and disease outcomes (28). And oxidative stress, identified as the primary pathogenic element in diabetes, represents a key initiator of endothelial dysfunction by diminishing nitric oxide levels via endothelial cell-selective adhesion molecule, which certified through mapping protein quantitative trait loci (29).

By elucidating the spectrum of characteristics attributed to each genetic locus, valuable insights can be gained in identifying loci that exert an impact on disease through shared mechanisms. This not only sheds light on expected associations among traits, but also reveals unforeseen connections. Undoubtedly, further investigations are imperative to establish conclusive statements regarding the roles of these loci. These investigations will encompass meticulous fine-mapping and colocalization methodologies, as well as the precise characterization of phenotypes associated with the relevant traits. Future extensive research on the potential repurposing of severe autoimmune T2DM for focal epilepsy treatment holds promise for providing invaluable understanding of the association between severe autoimmune T2DM and focal epilepsy (30). The present study has provided proof of the potential to identify alternate drugs with predicted efficacy if repurposed for epilepsy treatment.

This study has several limitations that should be considered. Firstly, the clinical diagnosis of severe autoimmune T2DM varied across different centers, which introduced the potential for inconsistencies. Secondly, the TSMR method employed in this study did not allow for the exploration of temporal effects. It remains unclear whether the epileptogenic process precedes, occurs simultaneously with, or follows the onset of severe autoimmune T2DM. Given recent evidence suggesting that seizures might predate severe hypoglycemia in severe autoimmune T2DM, further electrophysiological investigations are warranted in severe autoimmune T2DM patients at different disease stages. Thirdly, it is important to note that the participants in this study were of European ancestry. This focus limits the generalizability of our findings to other ethnic groups, as genetic backgrounds can vary significantly across different populations. The associations we identified between severe autoimmune T2DM and focal epilepsy may not fully capture the genetic diversity present in non-European populations. To assess the generalizability across diverse populations, future research utilizing GWAS data from other ethnicities, such as Biobank Japan and China Kadoorie Biobank, is essential. Expanding the research to include a broader range of genetic backgrounds will help determine whether the observed associations are consistent across different populations or if there are unique genetic factors at play in non-European groups. Furthermore, incorporating diverse populations into future studies will contribute to a better understanding of the global genetic architecture underlying severe autoimmune T2DM and focal epilepsy. This approach may also uncover population-specific variants and mechanisms that could be targeted for personalized medicine strategies.

Our study provides valuable insights into the potential causal relationship between severe autoimmune T2DM and focal epilepsy. These findings highlight the need for integrated management strategies that account for the bidirectional risks between these conditions. Future research should focus on examining these associations in diverse populations and investigating the underlying mechanisms through advanced genetic and electrophysiological studies. Additionally, our results suggest that severe autoimmune T2DM may be a modifiable risk factor for focal epilepsy, underscoring the importance of early identification and management of at-risk patients. The potential for repurposing existing drugs for the treatment of focal epilepsy presents a promising avenue for future therapeutic development.

The implications of these findings are significant for both research and clinical practice. Future studies should aim to validate our results across different ethnicities and explore the potential for personalized medicine approaches in managing patients with coexisting severe autoimmune T2DM and epilepsy. Moreover, clinicians should be aware of the increased risk of epilepsy in patients with severe autoimmune T2DM and consider incorporating epilepsy screening into routine care for these individuals.

## Conclusion

In summary, our findings emphasize the importance of future research aimed at prevention and targeted management strategies for patients with severe autoimmune T2DM and focal epilepsy. These approaches could enhance patient care, reduce the burden of epilepsy, and improve long-term outcomes for individuals affected by these interconnected conditions.

## Data Availability

The original contributions presented in the study are included in the article/**Supplementary Material**. Further inquiries can be directed to the corresponding authors.
